# Supplementary material for: Fluvoxamine maleate alleviates amyloid-beta load and neuroinflammation in 5XFAD mice to ameliorate Alzheimer disease pathology
Source: Front Immunol. 2024 Jul 29;15:1418422. doi: 10.3389/fimmu.2024.1418422 (PMC11317275; doi:10.3389/fimmu.2024.1418422)
Supplement: Supplementary file 1 [file Image_1.pdf]

Fluvoxamine maleate alleviates amyloid-beta load and neuroinflammation in 5XFAD mice to ameliorate Alzheimer disease pathology

General Scheme for the synthesis of Fluvoxamine maleate

The synthesis of fluvoxamine maleate was done by a well established method comprising of three steps with 5-methoxy-1-(4-(trifluoromethyl)phenyl)pentan-1-one as a starting material (**I**). In the first step compound (**I**) undergoes hydroxylation to form oxime (**II**) as an intermediate. The oxime (**II**) was then alkylated to give fluvoxamine base (**III**) which then undergoes salt formation with maleic acid yielding fluvoxamine maleate (**IV**) as the final desired product [1, 2]. The schematic representation for the synthesis of fluvoxamine maleate (**IV**) is shown below:

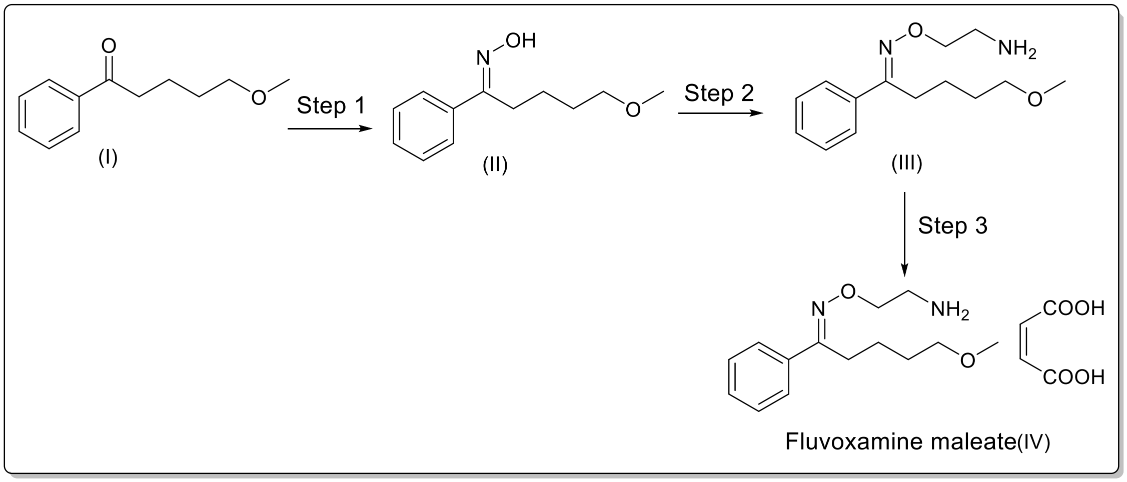

Scheme 1. Synthesis of Fluvoxamine maleate.

Spectra of Compounds

^{1}H NMR of Fluvoxamine maleate: ^{1}H NMR (400 MHz, DMSO-d^6) δ 7.80 (d, J= 8Hz, 2H), 7.61(d, J= 8Hz, 2H), 6.18(s, 2H) 4.48-4.36 (m, 2H), 3.28-3.22 (m, 11H), 2.81(m, 2H), 1.52(m, 4H); ^{19}F NMR (400 MHz, DMSO-d^6) -62.78.

^{1}H NMR of Fluvoxamine maleate

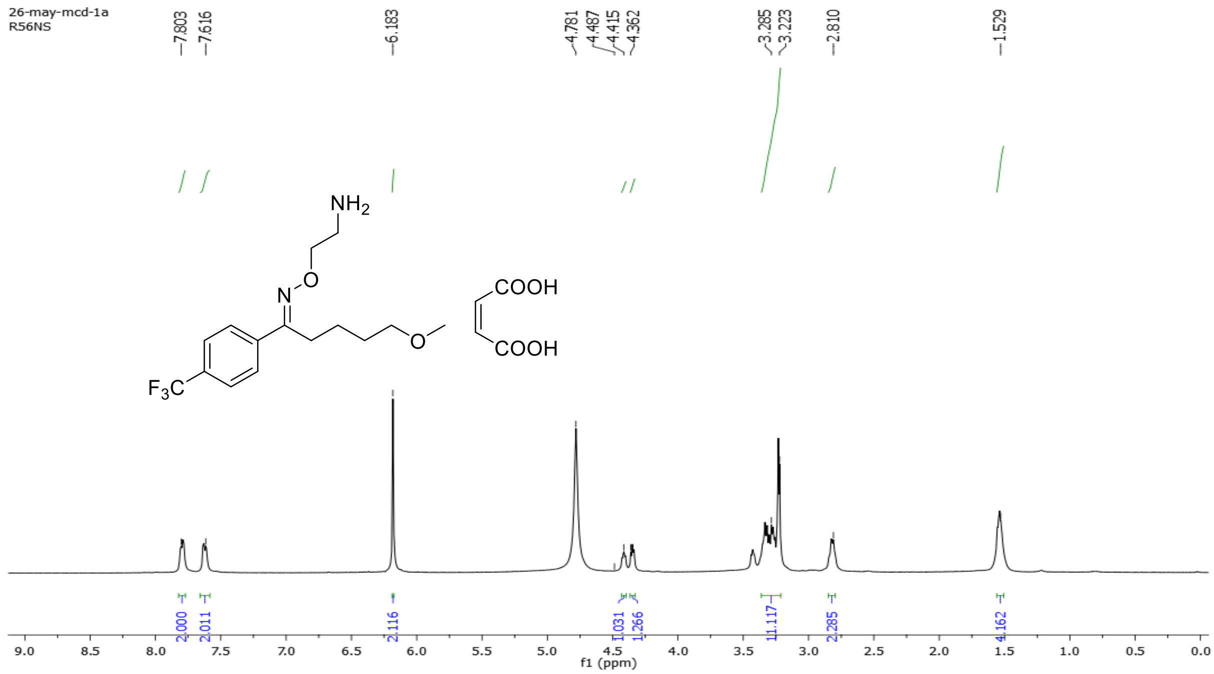

^{19}F NMR Spectra of Fluvoxamine maleate

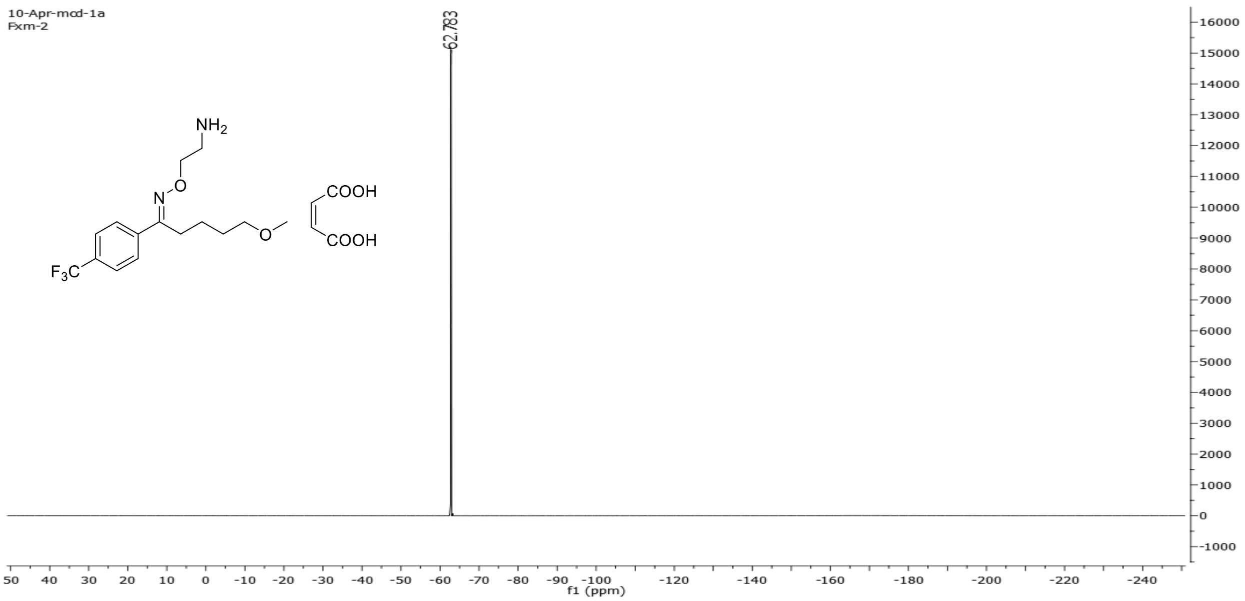

Figure S1

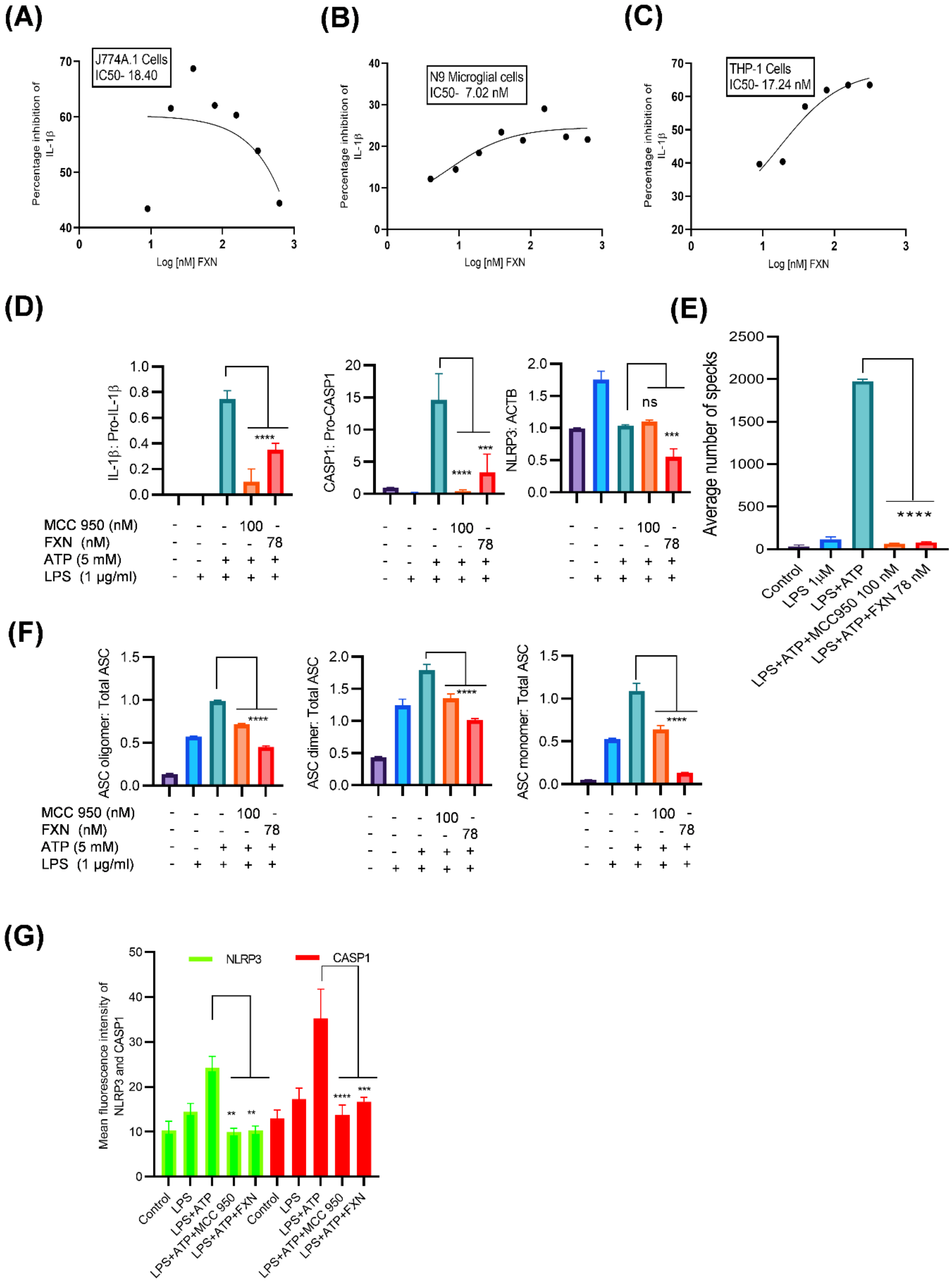

(A)
J774A.1 Cells
IC50- 18.40
Percentage inhibition of IL-1β
Log [nM] FXN
(B)
N9 Microglial cells
IC50- 7.02 nM
Percentage inhibition of IL-1β
Log [nM] FXN
(C)
THP-1 Cells
IC50- 17.24 nM
Percentage inhibition of IL-1β
Log [nM] FXN
(D)
IL-1β: Pro-IL-1β
CASP1: Pro-CASP1
NLRP3: ACTB
ns
MCC 950 (nM)
FXN (nM)
ATP (5 mM)
LPS (1 µg/ml)
(E)
Average number of specks
Control
LPS 1uM
LPS+ATP
LPS+ATP+MCC950 100 nM
LPS+ATP+FXN 78 nM
(F)
ASC oligomer: Total ASC
ASC dimer: Total ASC
ASC monomer: Total ASC
MCC 950 (nM)
FXN (nM)
ATP (5 mM)
LPS (1 µg/ml)
(G)
NLRP3
CASP1
Mean fluorescence intensity of NLRP3 and CASP1
Control
LPS
LPS+ATP
LPS+ATP+MCC 950
LPS+ATP+FXN

Figure S1: Analysis of effect of FXN on the NLRP3 inflammasome related protein expression in primary astrocytes

Graphs representing the (A) IC50 value of FXN in J774A.1 cells (B) IC50 value of FXN in N9 microglial cells (C) IC50 value of FXN in THP-1 cells. (D) Densitometric analysis of immunoblots in **Figure 1D**- IL-1β: Pro-IL-1β, CASP1: Pro-CASP1, NLRP3: ACTB**.** (E) Graph representing average number of specks in primary astrocytes shown in **Figure 1E**. (F) Densitometric analysis of immunoblots shown in **Figure 1F**- ASC oligomer, ASC dimer and ASC monomer normalized with total ASC. (G) Graph representing the mean fluorescence intensities of NLRP3 and CASP1 in the images provided in **Figure 1G**. The statistical analysis was performed using one-way ANOVA, followed by post-hoc Bonferroni test. The p-value <0.05 was considered to be statistically significant with values assigned as ****p < 0.0001, ***p < 0.001, **p< 0.01 and ns, not significant.

Figure S2

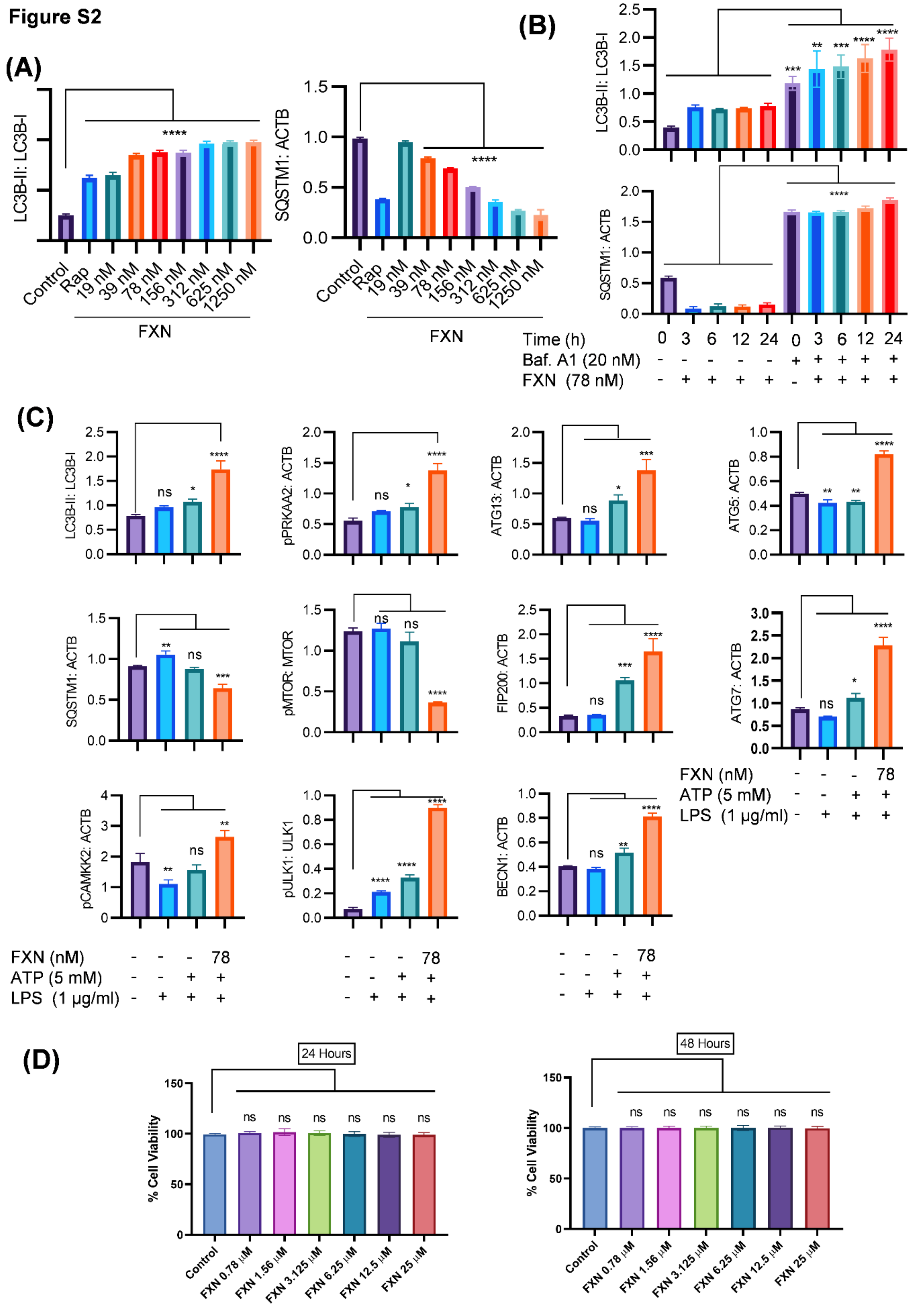

(A)
LC3B-II: LC3B-I
****
Control
Rap
19 nM
39 nM
78 nM
156 nM
312 nM
625 nM
1250 nM
FXN
SQSTM1: ACTB
1.5
1.0
0.5
0.0
****
FXN
(B)
LC3B-II: LC3B-I
2.5
2.0
1.5
1.0
0.5
0.0
***
**
***
****
****
SQSTM1: ACTB
2.0
1.5
1.0
0.5
0.0
****
Time (h) 0 3 6 12 24 0 3 6 12 24
Baf. A1 (20 nM) - - - - - + + + + +
FXN (78 nM) - + + + + - + + + +
(C)
LC3B-II: LC3B-I
pPRKAA2: ACTB
ATG13: ACTB
ATG5: ACTB
SQSTM1: ACTB
pMTOR: MTOR
FIP200: ACTB
ATG7: ACTB
pCAMKK2: ACTB
pULK1: ULK1
BECN1: ACTB
ns
*
****
**
***
FXN (nM) - - - 78
ATP (5 mM) - - + +
LPS (1 μg/ml) - + + +
(D)
24 Hours
48 Hours
% Cell Viability
150
100
50
0
ns
Control
FXN 0.78 μM
FXN 1.56 μM
FXN 3.125 μM
FXN 6.25 μM
FXN 12.5 μM
FXN 25 μM

Figure S2: Quantitative data showing impact of FXN treatment on autophagy related proteins in primary astrocytes under inflammatory conditions

Graphs showing Densitometric analysis of immunoblots (A) shown in **Figure 3A**- LC3B-II and SQSTM1 (B) **Figure 3B-** LC3B-II and SQSTM1 (C) **Figure 3E and 3F** LC3B-II: LC3B-I, SQSTM: ACTB, pCAMKK2: ACTB, pPRKAA2: ACTB, pMTOR: MTOR, pULK1: ULK1, ATG13: ACTB, FIP200: ACTB, BECN1: ACTB, ATG5: ACTB, and ATG7: ACTB. (D) Graph representing the cell viability of FXN up to 48 h. The statistical analysis was performed using one-way ANOVA, followed by post-hoc Bonferroni test. The p-value <0.05 was considered to be statistically significant with values assigned as ****p < 0.0001, ***p < 0.001, **p< 0.01, *p< 0.05 and ns= not significant.

Figure S3

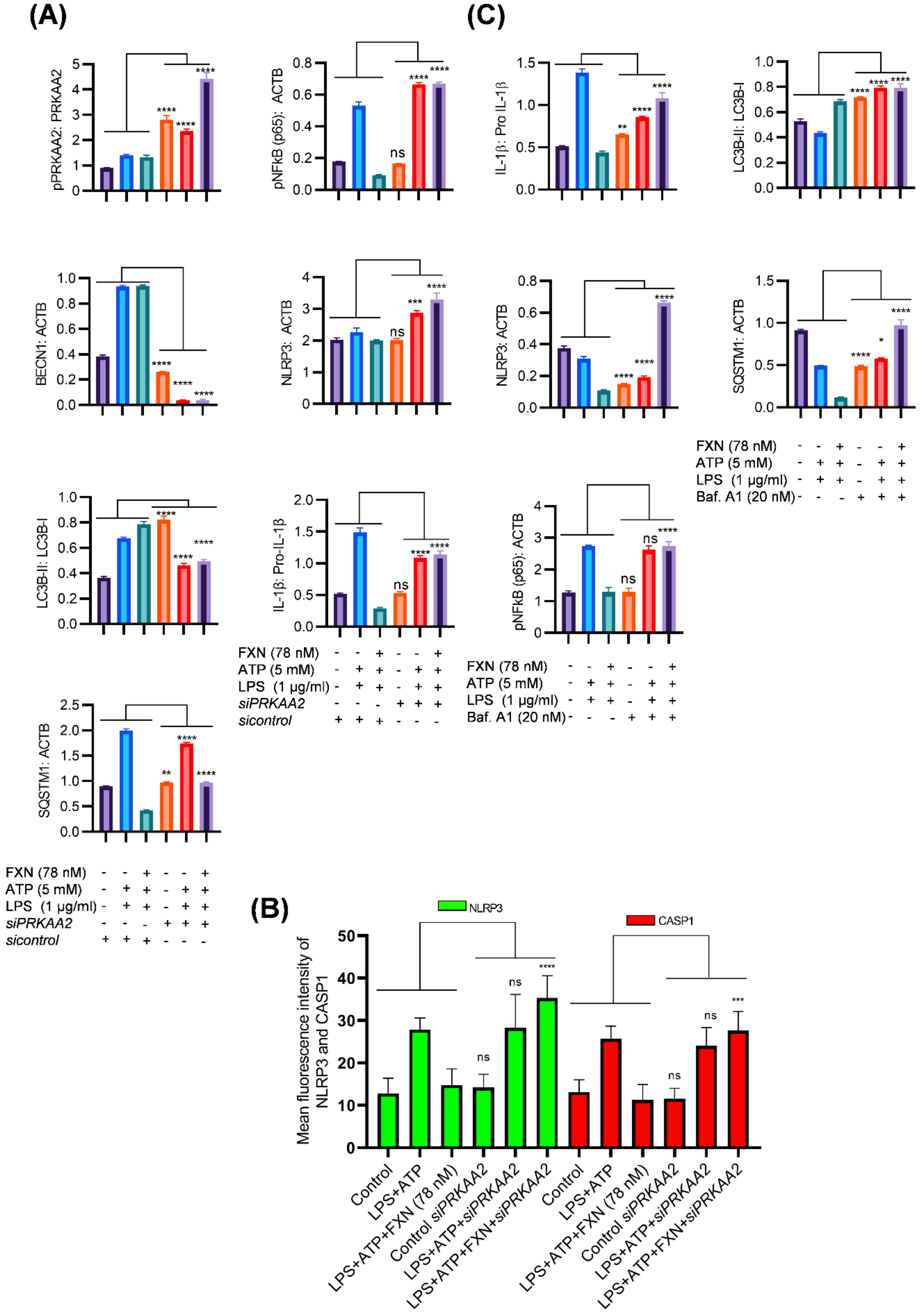

(A)
(C)
(B)
pPRKAA2: PRKAA2
pNFkB (p65): ACTB
IL-1β: Pro IL-1β
LC3B-II: LC3B-I
BECN1: ACTB
NLRP3: ACTB
SQSTM1: ACTB
IL-1β: Pro-IL-1β
pNFkB (p65): ACTB
FXN (78 nM)
ATP (5 mM)
LPS (1 µg/ml)
siPRKAA2
sicontrol
Baf. A1 (20 nM)
NLRP3
CASP1
Mean fluorescence intensity of NLRP3 and CASP1
Control
LPS+ATP
LPS+ATP+FXN (78 nM)
Control siPRKAA2
LPS+ATP+siPRKAA2
LPS+ATP+FXN+siPRKAA2

Figure S3: Analysis of effect of FXN on NLRP3 inflammasome via modulating autophagy

(A) Densitometric analysis of immunoblots given in **Figure 4A-** pPRKAA2: ACTB, BECN1: ACTB, LC3B-II: LC3B-I, SQSTM: ACTB, pNF-κB (p65): ACTB, IL-1β: Pro-IL-1β and NLRP3: ACTB. (B) Graph depicting the mean fluorescence intensities of NLRP3 and CASP1 in the images provided in **Figure 4B**. (C) Densitometry of immunoblots provided in **Figure 4D**- IL-1β: Pro-IL-1β, NLRP3: ACTB, pNF-κB (p65): ACTB, LC3B-II: LC3B-I, SQSTM: ACTB. The statistical analysis was performed using one-way ANOVA, followed by post-hoc Bonferroni test. The p-value <0.05 was considered to be statistically significant with values assigned as ****$p < 0.0001$, ***$p < 0.001$, **$p< 0.01$, *$p< 0.05$ and ns= not significant.

Figure S4

(A)

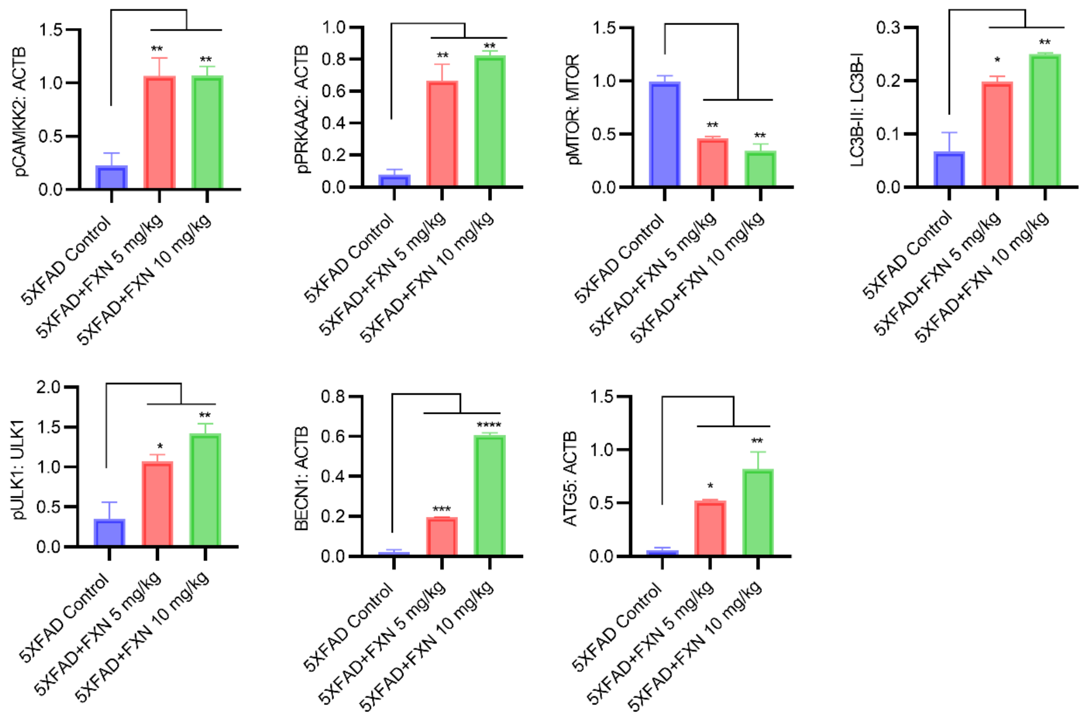
pCAMKK2: ACTB
pPRKAA2: ACTB
pMTOR: MTOR
LC3B-II: LC3B-I
pULK1: ULK1
BECN1: ACTB
ATG5: ACTB
5XFAD Control
5XFAD+FXN 5 mg/kg
5XFAD+FXN 10 mg/kg

(B)

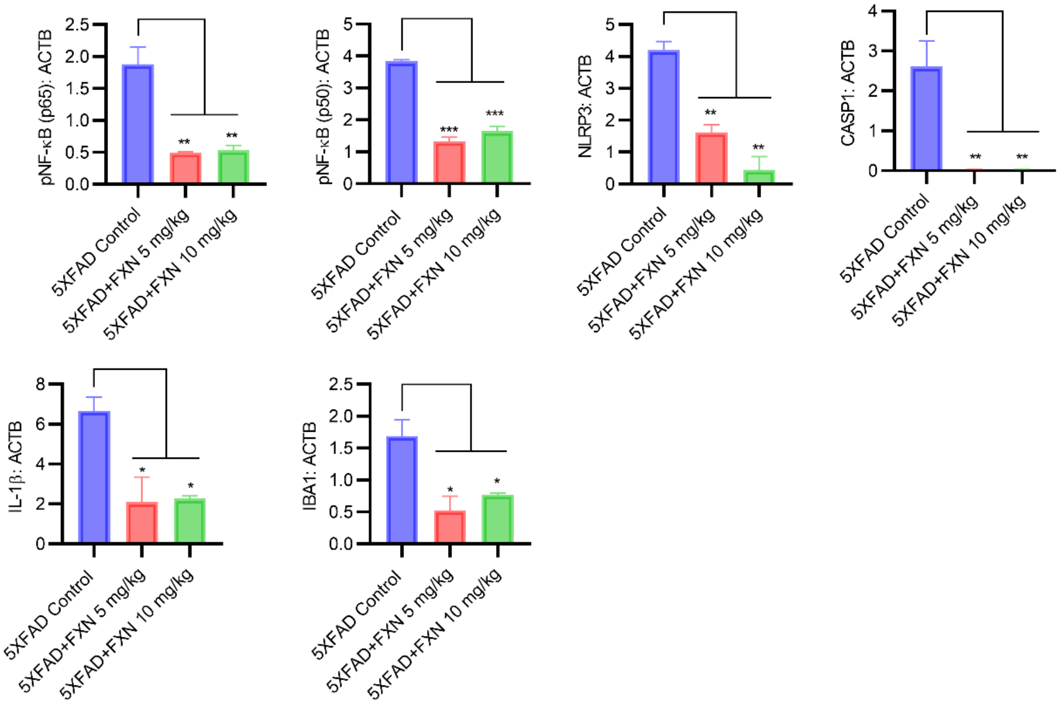
pNF-κB (p65): ACTB
pNF-κB (p50): ACTB
NLRP3: ACTB
CASP1: ACTB
IL-1β: ACTB
IBA1: ACTB
5XFAD Control
5XFAD+FXN 5 mg/kg
5XFAD+FXN 10 mg/kg

Figure S4: Quantitative data showing the effect of FXN on autophagic and inflammatory proteins in hippocampal region of 5XFAD mice

Densitometric analysis of immunoblots (A) shown in **Figure 8B-** pCAMKK2: ACTB, pPRKAA2: ACTB, pMTOR: MTOR, LC3B-II: LC3B1, pULK: ULK1, BECN1: ACTB and ATG5: ACTB (B) **Figure 8C-** NF-κB (p65), NF-κB (p50), NLRP3, CASP1, IL-1β and IBA1 normalized with ACTB. The statistical analysis was performed using one-way ANOVA, followed by post-hoc Bonferroni test. The p-value <0.05 was considered to be statistically significant with values assigned as ****p < 0.0001, ***p < 0.001, **p< 0.01, *p< 0.05 and ns= not significant.
